# Association Between Smartphone Addiction and Academic Performance Among Rural School Students of Telangana

**DOI:** 10.7759/cureus.113058

**Published:** 2026-07-20

**Authors:** Pratik R Sonunkar, Bhushan Kamble, G Alekhya, Govindrao N Kusneniwar, Sandip Dhole, Enubothula Sampath Kumar, Gayatri Putukuchi

**Affiliations:** 1 Community and Family Medicine, All India Institute of Medical Sciences, Hyderabad, IND; 2 Physical Medicine and Rehabilitation, All India Institute of Medical Sciences, Hyderabad, IND; 3 Community Medicine, All India Institute of Medical Sciences, Hyderabad, IND

**Keywords:** academic performance, adolescents, india, rural schools, smartphone addiction

## Abstract

Introduction

The widespread use of smartphones among adolescents has raised concerns about behavioural addiction and its potential academic consequences. However, evidence from rural Indian school-going adolescents is scarce. The current study aims to estimate the prevalence of smartphone addiction and evaluate its association with academic performance among rural adolescents in Telangana.

Methods

A cross-sectional study was conducted among 423 students aged 10-19 years from three randomly selected government schools in the Rural Health Training Centre catchment area. Data on sociodemographics and smartphone use were collected using a pre-tested questionnaire. Smartphone addiction was measured using the Smartphone Addiction Scale-Short Version. The students' academic performance was obtained from school records. Statistical analysis included the chi-square test, t-test, and logistic regression.

Results

The mean age of participants was 14.0 ± 1.33 years. A total of 85.8% (363) participants reported smartphone use, with 32.2% (117) having smartphone addiction. No significant correlation or association was found between smartphone addiction and academic performance (Spearman's rho = 0.06, p=0.178). Smartphone addiction was associated with other variables such as male gender, personal ownership of a smartphone, poor sleep quality, and initiation of smartphone use before the COVID-19 pandemic in multivariable logistic regression analysis.

Conclusion

Smartphone addiction was prevalent in one-third of rural school-going adolescents and was associated with factors such as male sex, personal smartphone ownership, initiation of use before COVID-19, and poor sleep quality. Our study findings identify factors for school-based awareness programmes and further longitudinal research to promote healthy digital habits among rural adolescents.

## Introduction

The widespread use of smartphones has transformed daily life, reshaping communication, education, and entertainment. By 2021, global smartphone users were estimated at >3.8 billion [1]. In 2022, the total number of smartphone users was 6.25 billion, with India contributing 659 million users, second only to China’s 974 million [2]. Adolescents are among the most active user groups, as smartphones serve multiple functions, including educational access, social networking, entertainment, and communication. As dependence on smartphones grows, concerns have emerged regarding their excessive use and potential for addiction among adolescents [3,4].

While smartphones offer several advantages for learning and connectivity, excessive use has raised concerns regarding behavioural addiction. Smartphone addiction is recognised as a behavioural disorder, characterised by compulsive use, mood alterations, tolerance, dependence, and functional impairment [5-7]. This pattern of problematic use affects not only physical and mental health but also social functioning and emotional well-being. A growing body of literature highlights the association between smartphone addiction and impaired sleep [8-10]. Excessive screen time, late-night usage, and constant connectivity are associated with disturbed sleep patterns, shorter sleep duration, and poor sleep quality [11-13]. Such disruptions can negatively affect attention, memory, and emotional regulation, undermining adolescent health and development.

Adolescence is a critical developmental period marked by significant cognitive, emotional, and behavioural transitions. Excessive smartphone use during this stage may influence attention span, self-regulation, and learning behaviour. Previous studies have reported that more than one-third of participants exhibit features of smartphone addiction [14,15]. The existing literature has assessed smartphone use among urban populations or medical and university students, while rural school-going adolescents remain underrepresented [16,17]. In addition to health-related effects, smartphone addiction may also influence students’ academic performance. Excessive smartphone use can lead to reduced study time, distraction during learning activities, decreased concentration, and procrastination, all of which may negatively affect academic achievement. Frequent engagement with social media, gaming, and entertainment applications may divert attention away from academic responsibilities, thereby impairing learning outcomes and overall scholastic performance [18,19].

A systematic review and meta-analysis reported that the prevalence of smartphone addiction among Indian adolescents ranges from 39% to 44% [20]. Previous evidence has consistently demonstrated that smartphone addiction is associated with poorer academic performance and reduced attention among adolescents [21]. However, most published studies in India have been conducted among urban or mixed populations, while evidence specific to rural school adolescents remains limited. Rural adolescents differ from their urban counterparts in terms of socioeconomic conditions, digital infrastructure, educational resources, parental supervision, and patterns of smartphone ownership and use, all of which may influence both the prevalence of smartphone addiction and its impact on academic performance [22]. Consequently, findings from urban populations may not be directly generalisable to rural settings. We therefore hypothesised that smartphone addiction is not associated with academic performance among rural school students in Telangana. Accordingly, the present study aimed to estimate the prevalence of smartphone addiction and examine its association with academic performance among rural school students in Telangana. The findings are expected to provide context-specific evidence that may inform school-based interventions and public health strategies to promote responsible smartphone use and healthier digital habits among rural adolescents.

## Materials and methods

Study design and setting

This community-based cross-sectional study was conducted in the rural field practice area of the Department of Community Medicine, All India Institute of Medical Sciences (AIIMS), Bibinagar, Telangana, India. The department provides comprehensive primary health care services through its Rural Health Training Centre (RHTC) located in a Bommalaramaram mandal of Telangana. The Bommalaramaram mandal has 15 government high schools. All government schools operate on a standard curriculum, with infrastructure and student evaluation in accordance with guidelines issued by the Government of Telangana. From these, three schools were selected by lottery (simple random sampling). The study was conducted from November 2023 to April 2024, while data collection in the selected schools was undertaken over a 60-day period between January and February 2024.

Study population

The sample size was calculated using a prevalence estimate of 24.7% from a previous study [23] and the formula for cross-sectional studies, 

$$n=\frac{Z_{1-\alpha/2}^{2}P(1-P)}{d^{2}}$$

where Z₁₋α/₂=1.96 (at 95% confidence level), p=0.247 (prevalence from a previous study), and d=0.05 (absolute precision of 5%), yielding a minimum sample size of 297. Allowing for a 15% non-response rate, the target sample size was 341, rounded to 350.

**Figure 1:**
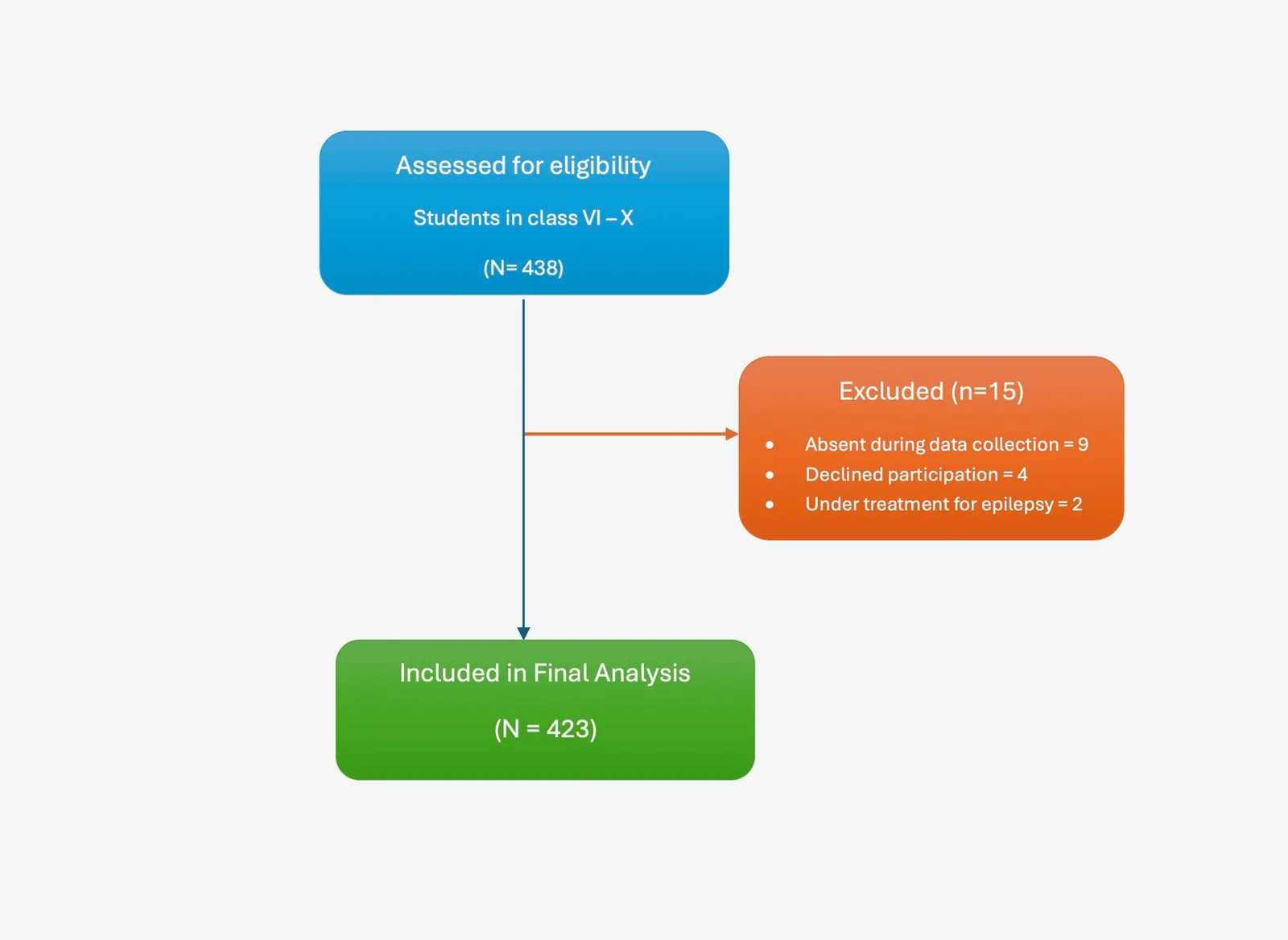
Flowchart of enrolment of study participants

From each selected school, students studying in classes VI-X were included using a complete enumeration approach. Exclusion criteria included students currently undergoing treatment for any psychiatric illness and those who were unable to comprehend the questionnaire after explanation by the investigators. A total of 438 students enrolled in classes VI-X were assessed for eligibility. Of these, 9 students were absent during data collection, 4 declined participation, and 2 met the exclusion criteria (under treatment for epilepsy), resulting in 423 students included in the final analysis. Although the minimum required sample size was calculated as 350, a complete enumeration strategy was adopted. Therefore, all eligible students from classes VI-X in the three selected schools who fulfilled the inclusion criteria were invited to participate, resulting in a final sample of 423 students (Figure 1). 

Study procedure

Assent was obtained from students, and their parents/guardians provided written informed consent to participate. Comprehension was assessed by asking students to read the questionnaire instructions and explain the meaning of two sample questions in their own words before participation. Students were considered unable to comprehend the questionnaire if they failed to understand the study instructions or correctly explain the meaning of the sample questions, despite the investigators' clarification.

The questionnaire was pilot-tested on 20 students from a school not included in the main study to assess clarity, comprehensibility, and completion time. During pilot testing, students were specifically asked whether they understood each questionnaire item. Minor wording modifications were made to improve clarity. Because English is a compulsory subject in the participating schools, and students demonstrated adequate comprehension during pilot testing, a translated version was not considered necessary. Students were administered a pre-tested semi-structured questionnaire privately without the presence of school staff. It consisted of sociodemographic details, the Smartphone Addiction Scale-Short Version (SAS-SV), the Pittsburgh Sleep Quality Index (PSQI), and academic records collected from the schools [24]. Before administering the questionnaire, students were briefed that their responses would be anonymous and that information would not be shared with teachers or parents. We defined smartphone use as “use of any smartphone during 24 hours by the student for any purpose.” Participants also reported their average daily duration of smartphone use, categorised as ≤10 minutes, 11-60 minutes, 1-2 hours, 3-4 hours, 5-6 hours, and >6 hours. Smartphone addiction was assessed using the SAS-SV, a validated 10-item scale rated on a six-point Likert scale from 1 (strongly disagree) to 6 (strongly agree). A total score of ≥31 for boys and ≥33 for girls indicated smartphone addiction. For assessment of sleep quality, if the overall PSQI score was ≤5, it was defined as good sleep quality, and if the PSQI score was >5, it was defined as poor sleep quality [25]. Academic performance was measured using marks from the last three semester examinations, with data extracted from school records to minimise recall bias. Academic performance was categorised as good if the average of the previous three exam scores was ≥60% and poor if <60%. This cut-off was considered based on the grading system employed by the Department of Education, Telangana State, for government schools. In addition, attendance records of the selected students were obtained from the school offices to supplement the academic performance evaluation. The SAS-SV is validated and freely available in the public domain. Screen time of <2 hours was considered in line with recommendations from the Indian Academy of Pediatrics [26].

Statistical analysis

Data were entered into Microsoft Excel 2023 (Microsoft Corp., Redmond, WA, USA) and analysed using IBM SPSS Statistics version 22 (IBM Corp., Armonk, NY, USA). Descriptive statistics were presented as means, standard deviations, and proportions as appropriate. The chi-square test assessed associations between smartphone addiction and sociodemographic variables. Student’s t-test was used for continuous variables. Variables with p-values <0.20 in the univariable analysis and those considered epidemiologically relevant based on previous literature were entered into the multivariable logistic regression model. Multicollinearity among independent variables was assessed using the variance inflation factor (VIF), and no evidence of significant multicollinearity was observed (all VIF values <2). Model calibration was assessed using the Hosmer-Lemeshow goodness-of-fit test, which indicated an adequate model fit (p>0.05). Data completeness was checked at the time of collection, and no missing data were present in the final analysis. A p-value <0.05 at 95% confidence intervals was considered statistically significant.

Ethical approval

The study received approval from the Institute’s Research Cell and the Institutional Ethics Committee of the AIIMS, Bibinagar. Permission was obtained from the school principals to conduct the study. Written informed consent was obtained from parents/guardians for participants aged 18 years or younger, and assent was obtained from those aged 12-17 years. Confidentiality was maintained, and academic and attendance records were used solely for research. Students identified with smartphone addiction were counselled and referred to the psychiatrist at the Institute's RHTC for further evaluation, where considered appropriate. SAS-SV was used with written permission from its first author, Mr Min Kwon (Korea).

## Results

In the present study, out of 423 participants, the majority were studying in 10th class (34.9%), and 69.5% had attendance of more than 75% in the last three months. The majority (77.3%) had academic performance of ≥60%, which was categorised as satisfactory (Table 1).

**Table 1:** Distribution of study participants as per academic details (N=423)

Variable		n (%)
Class	6	21 (4.9)
	7	32 (7.7)
	8	101 (23.9)
	9	121 (28.6)
	10	148 (34.9)
Attendance in school	<75%	129 (30.5)
	≥75%	294 (69.5)
Academic performance	<60	96 (22.7)
	≥60	327 (77.3)

Of 423 adolescents, 363 (85.8%) reported using smartphones, the majority (67.8%) initiating use after the COVID-19 pandemic. Out of 363 students who have used smartphones, 117 (32.2%) had smartphone addiction. Daily duration of use was most commonly 1-2 hours (62.8%), while 2.5% reported more than 6 hours. Entertainment (59.5%), social media (66.4%), and gaming (46.0%) were the most common reasons for using smartphones (Table 2).

**Table 2:** Distribution of study participants as per smartphone use (N=363) *Multiple responses possible.

Variable		n (%)
Use a smartphone? (N=423)	Yes	363 (85.8)
	No	60 (14.2)
Duration of daily smartphone use (n=363)	10 min	27 (7.4)
	11-16 min	33 (9.1)
	1-2 h	228 (62.8)
	3-4 h	42 (11.6)
	5-6 h	24 (6.6)
	>6 h	9 (2.5)
Use of smartphones* (n=363)	Educational purpose	171 (47.1)
	Entertainment	216 (59.5)
	Social media	241 (66.4)
	Gaming	167 (46.0)
	Listening music	101 (27.8)
	Calling and chatting	155 (42.7)
If using for educational purposes, then for what* (n=171)	Online lectures	19 (11.1)
	Online exams	58 (33.9)
	Reading	38 (22.2)
	YouTube video	66 (38.6)
	Question practice	43 (25.1)
	Learning skills	43 (25.1)

In the present study, we examined the association between smartphone addiction, academic performance, and sociodemographic factors. Table 3 shows that smartphone addiction was significantly associated with age, sex, attendance in school, personal smartphone, duration of use, usage status in relation to COVID-19, and sleep quality (p<0.01). 

**Table 3:** Factors associated with smartphone addiction and different variables (N=363) *p≤0.05 is considered statistically significant. Chi-square test was used for categorical variables; student’s t-test was applied for continuous variables (age). The test statistic column shows chi-square (χ²) values for categorical variables and t-values for continuous variables. PSQI, Pittsburgh Sleep Quality Index.

Variable		Smartphone addiction		Test statistic value	p-value
		Yes (n=117)	No (n=246)		
Age in years, mean (SD)		14.5 (1.18)	13.8 (1.35)	4.28	<0.001*
Sex	Male	80 (46.5)	92 (37.4)	29.75	<0.001*
	Female	37 (31.5)	154 (62.6)		
Attendance in school	<75%	47 (40.18)	65 (26.5)	11.97	0.001*
	≥75%	70 (59.82)	181 (73.5)		
Academic performance	<60%	16 (13.6)	57 (23.7)	0.47	0.491
	≥60%	101 (86.4)	189 (65.3)		
Personal smartphone	Yes	44 (37.6)	39 (15.8)	20.81	<0.001*
	No	73 (62.3)	207 (84.2)		
Duration of smartphone use	<2 h	73 (62.3)	215 (87.3)	25.63	<0.001*
	≥2 h	44 (37.7)	31 (12.7)		
Father’s education	Literate	89 (76.1)	186 (75.9)	0.124	0.725
	Illiterate	28 (24.9)	60 (24.1)		
Mother’s education	Literate	73 (57.5)	184 (74.7)	1.06	0.304
	Illiterate	54 (42.5)	62 (25.3)		
Sleep quality	Good (PSQI≤5)	33 (25.9)	174 (70.8)	38.7	<0.001*
	Poor (PSQI>5)	94 (74.1)	72 (29.2)		
Before COVID-19		49 (41.8)	68 (27.6)	12.45	<0.001*
After COVID-19		68 (58.2)	178 (72.4)		

There was no correlation between smartphone addiction and academic scores of the study participants (Spearman’s rho = 0.06, p-value = 0.178). A significant association was observed between smartphone users and non-users regarding academic performance (χ²=5.46, p=0.019).

Male sex (adjusted odds ratio (aOR): 1.93 (1.04-2.12), p<0.001), beginning smartphone use before COVID-19 (aOR: 2.45 (1.43-4.08), p=0.001), personal smartphone ownership (aOR: 2.71 (1.45-5.06), p=0.002), and good sleep quality (aOR: 0.28 (0.16-0.47), p<0.001) were independently associated with smartphone addiction. Late adolescence, low attendance, and screen time duration, though significant in the unadjusted analysis, lost significance after adjusting for confounders (Table 4).

**Table 4:** Bivariate and multivariate logistic regression analysis of factors associated with smartphone addiction *p≤0.05 is considered statistically significant. Logistic regression was used to compute unadjusted and adjusted ORs. Model fit: Overall model χ²=76.2 (df=10), p<0.001. McFadden's R²=0.168, indicating acceptable model fit. Multicollinearity: Variance inflation factor ranged from 1.04 to 1.33 for all predictor variables. Model calibration: Hosmer–Lemeshow goodness-of-fit test p>0.05, indicating adequate model calibration. CI, Confidence interval; OR, Odds ratio.

Factor	Unadjusted OR (95% CI)	p-value	Adjusted OR (95% CI)	p-value
Late adolescents	1.68 (1.08-2.60)	0.02*	0.88 (0.52-1.52)	0.650
Male sex	3.55 (2.27-5.64)	<0.001*	1.93 (1.04-2.12)	<0.02*
Began smartphone use before COVID	2.35 (1.48-3.75)	<0.001*	2.45 (1.43-4.08)	0.001*
Screen time (≥2 h)	4.94 (2.93-8.40)	<0.001*	1.17 (0.65-2.09)	0.604
Satisfactory academic performance	1.27 (0.76-2.18)	0.377	1.95 (0.96-3.94)	0.065
Owned personal smartphone	3.38 (2.01-5.71)	<0.001*	2.71 (1.45-5.06)	0.002*
Low attendance	2.20 (1.41-3.44)	<0.001*	0.63 (0.37-1.08)	0.093
Literate father	0.92 (0.58-1.45)	0.725	0.97 (0.52-1.67)	0.937
Literate mother	0.80 (0.52-1.22)	0.304	0.93 (0.56-1.50)	0.796
Good sleep quality	0.24 (0.15-0.39)	<0.001*	0.28 (0.16-0.47)	<0.001*

## Discussion

The present study assessed the prevalence of smartphone addiction and its association with sociodemographic factors, smartphone use behaviours, and sleep quality among rural school-going adolescents in Telangana. The findings demonstrate that smartphone use is highly prevalent among adolescents, with a considerable proportion having smartphone addiction.

In the current study, 85.8% of participants reported using smartphones. This high prevalence reflects the rapid digital penetration even in rural settings. Similar levels of smartphone accessibility among adolescents have been reported in previous studies conducted in India [27]. The smartphone use observed in this study after the COVID-19 pandemic is consistent with findings from other studies reporting increased smartphone use following the transition to online learning and digital communication during the pandemic [28]. Among users, 20.7% reported daily usage exceeding two hours, comparable to figures from studies in Gujarat and Navi Mumbai [14,29]. The prevalence of smartphone addiction was 32.2%. This finding is comparable to reports from studies conducted in India and other countries [2,20]. Male students in our sample exhibited a higher prevalence of smartphone addiction compared with females, which was similar to other studies in Navi Mumbai, where male students had more mobile phone dependence [29]. 

In multivariable logistic regression analysis, male sex, personal smartphone ownership, initiation of smartphone use before the COVID-19 pandemic, and poor sleep quality emerged as independent predictors of smartphone addiction. Similar findings have been reported in other studies, which also identified demographic and behavioural factors, such as age, gender differences, patterns of smartphone use, and psychosocial influences, as significant determinants of smartphone addiction among adolescents [30-33]. Students who have started using smartphones before the onset of the pandemic must have increased their use following the pandemic, which would have led to smartphone addiction. In our study, no correlation was found between academic scores and smartphone addiction scores. Furthermore, academic performance was not independently associated with smartphone addiction after adjustment for potential confounding variables in the multivariable logistic regression analysis. These findings support our study hypothesis that smartphone addiction would not be significantly associated with academic performance among rural school-going adolescents. Academic achievement is a multifactorial outcome influenced by factors such as individual learning ability, school attendance, quality of teaching, parental support, socioeconomic circumstances, and study habits, which may have a greater influence on academic performance than smartphone use alone. However, other studies have shown that smartphone use negatively impacts academic outcomes [34-36]. Good sleep quality was strongly associated with reduced odds of smartphone addiction, suggesting a protective effect. Similar findings were noted in other Indian studies that adolescents and college students with problematic smartphone use were significantly more likely to experience poor sleep quality and sleep disturbances [37,38]. These findings highlight the importance of promoting healthy smartphone use and sleep hygiene among adolescents.

The strengths of this study include the relatively large sample size, the use of a validated instrument (SAS-SV) to assess smartphone addiction, and the use of school academic records, which reduced the risk of recall bias. Furthermore, this study contributes important evidence regarding smartphone addiction among rural school-going adolescents, a population that has been relatively underrepresented in previous research. However, several limitations should be considered while interpreting the findings. First, the cross-sectional design limits the ability to establish causal relationships between smartphone addiction and sociodemographic characteristics. Second, the study was conducted in government schools within a single rural mandal, which may limit the generalisability of the findings. A systematic review and meta-analysis assessing smartphone addiction among Indian medical students highlighted several methodological issues, including that prevalence varied across scales, study populations, and settings [39]. Third, smartphone usage patterns were self-reported and therefore may be subject to reporting bias. Parental assessments were not collected for cross-validation, potentially leading to misclassification of actual smartphone use. Fourth, several important potential confounders were not assessed in this study, including socioeconomic status, mental health status, family environment, and students' baseline academic scores. 

## Conclusions

This study demonstrates a high prevalence of smartphone addiction among rural school-going adolescents in Telangana. Smartphone addiction was significantly associated with various demographic, social, and behavioural factors such as male sex, owning a personal smartphone, beginning smartphone use before COVID-19, and sleep quality. These findings highlight the need for further longitudinal research and suggest that targeted awareness programmes involving teachers, parents, and school health teams addressing both digital habits and sleep hygiene, with particular attention to gender-sensitive approaches, may be warranted as a policy consideration to promote healthy development among rural adolescents.

## Appendices

A. Sociodemographic details:

1. Name:

2. Age in years:

3. Sex:

4. DOB:

5. Religion:

6. Address:

7. Mobile number:

8. Father’s education:

9. Father’s occupation:

10. Mother’s education:

11. Mother’s occupation:

12. Total family income:

13. No. of siblings:

14. Weight:

15. Height:

B. Academic details:

1. School name and address:

2. Roll no:

3. Total attendance:

4. Academic performance in the last three exams:

Semester 1

Semester 2

Semester 3

Average

Marks

C. Smartphone-related information:

1. Do you use a smartphone?

a) Yes

b) No 

2. If yes, is it your personal smartphone?

a) Yes

b) No

3. When did you start using a smartphone?

a) Before COVID

b) After COVID

4. Duration of smartphone use/screen time on a typical day:

a) <10 min 

b) 11-16 min

c) 1-2 h

d) 3-4 h

e) 5-6 h

f) >6 h

5. Frequency of use on a typical day:

a) <5 times/day

b) 6-10 times/day 

c) 10-15 times/day

d) >15 times/day

6. First screen exposure in the early morning is within the duration of:

a) <1 min

b) <5 min

c) <15 min

d) <30 min

e) <1 h

f) >1 h

7. Use of smartphones is for:

a) Educational purpose

b) Entertainment

c) Social media

d) Gaming

e) Listening to music

f) Calling and chatting

8. If using for educational purposes, then for what?

a) Lecture

b) Exam

c) Reading

d) YouTube video

e) Question practice

f) Learning skills
